# Calibration of Deformable Mirrors for Open-Loop Control

**DOI:** 10.3390/s22218465

**Published:** 2022-11-03

**Authors:** Marcel Leutenegger, Stefan W. Hell

**Affiliations:** Department of NanoBiophotonics, Max Planck Institute for Multidisciplinary Sciences, Am Faßberg 11, 37077 Göttingen, Germany

**Keywords:** off-axis interferometry, phase unwrapping, deformable mirror control

## Abstract

Deformable mirrors enable the control of wave fronts for the compensation of aberrations in optical systems and/or for beam scanning. Manufacturers of deformable mirrors typically provide calibration data that encode for the fabrication tolerances among the actuators and mirror segments to support open-loop control with high wave front fidelity and accuracy. We report a calibration method that enables users of the deformable mirrors to measure the response of the mirror itself to validate and improve the calibration data. For this purpose, an imaging off-axis Michelson interferometer was built that allowed measuring the mirror topography with high accuracy and sufficient spatial resolution. By calibrating each actuator over its entire range, the open-loop performance for our deformable mirror was improved.

## 1. Introduction

The atmospheric turbulence in terrestrial astronomy [1,2] and free-space optical communication [3,4] or sample-induced wavefront aberrations in optical microscopy [5] can be corrected by mirrors whose surfaces are deformed by multiple actuators [6]. For quasi-stationary applications, the deformation of a mirror is typically controlled iteratively to maximize the optical performance of the system [1,2,3,4,5,7,8]. This closed-loop control requires means for measuring the wavefront and/or the performance of the optical system, and it might be too slow for applications that require rapid deformations of the mirror. For dynamic applications, the deformation of the mirror can be set directly without real-time verification of the mirror’s shape. Micro electro-mechanical system (MEMS) mirrors that show no hysteresis [6,9] are particularly suitable because open-loop control relies on calibration data measured beforehand. MEMS mirrors’ manufacturers usually provide typical displacement curves of the actuators and a map for the flat-field correction of the entire mirror [9]. Although these data may be sufficient for many applications, we required the calibration of each individual actuator over its full displacement range for the accurate control of the wavefront in a MINSTED microscope [10].

For this purpose, we built an off-axis Michelson interferometer to image the topography of a segmented mirror with high precision and accuracy, which means that we aimed at measurement errors of a few nanometers at most. Table 1 compares several techniques for measuring the wavefront of coherent light beams that we considered. A Shack–Hartmann sensor [11,12] measures the mean wavefront gradient on many small regions of the incident beam. The reconstitution of the mirror topography requires integration of the measured gradient [13] or analyzing the detected image iteratively [14] to limit the accumulation of measurement noise. Light field cameras [15] trade the precision of the wavefront gradient measurement with a higher sampling density and are more suitable for photographic imaging of rapidly varying scenes. In both cases, the wavefront sampling is rather coarse, and the gradient measurement hampers the quantification of absolute displacements. In contrast, full-field quantitative phase imaging in an off-axis common-path configuration [16] features high-resolution and phase-stable measurements. However, as it references the incident light field with a low-pass filtered copy of itself, it underreports the absolute displacement of large features whose phase variations pass through the filtering pinhole. This remaining limitation was addressed by an imaging Michelson interferometer with off-axis reference beam similar to the interferometer by Nejdl et al. [17].

During calibration, we measured the mirror topography at 256 sampling points spanning the entire actuator command voltages and extracted the displacement curves for each individual actuator. Finally, commanding the deformable mirror to various continuous shapes, we measured and validated the mirror topographies with respect to the target shapes and obtained residual errors within the vendor’s closed-loop control specification.

## 2. Materials and Methods

**Figure 1:**
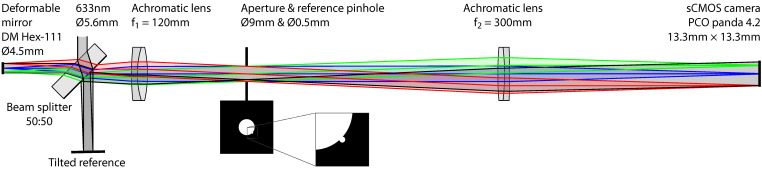
Imaging Michelson interferometer with tilted reference beam for heterodyne phase retrieval.

Figure 1 illustrates the off-axis imaging interferometer that was used to measure the mirror topography. A HeNe laser beam was expanded to about 10 mm diameter and truncated at 5.6 mm diameter. A 50:50 beam splitter directed this beam to a reference mirror and to the deformable mirror, whose active surface covered a circle of about Ø4.5 mm diameter. The reflected beams were recombined by the 50:50 beam splitter and imaged at 2.5× magnification by a Kepler telescope on the camera sensor. An aperture of 9 mm diameter in the common focal plane of the telescope lenses defined the numerical aperture of the imaging system. The reference mirror was tilted such that its reflected beam passed through the reference pinhole of 0.5 mm diameter 4.7 mm off-axis. The reference pinhole was rotated such that the interference pattern was modulated along a diagonal of the camera sensor at high spatial frequency. The scientific CMOS camera imaged a 5.3 mm × 5.3 mm region of the deformable mirror, which contained its active area and some of the surrounding inactive mirror elements.

A thick 10 mm BK7 substrate with a refraction index of 1.515 was chosen for the 50:50 beam splitter, and an angle of incidence of 45° was set up to spatially separate the reflections from its front and back surfaces. This choice eliminated perturbations of the interference pattern due to multiple reflections by the beam splitter’s faces, but a strong astigmatism was introduced with a root mean square (RMS) wavefront aberration of 0.8 wavelengths. The spatial resolution of the mirror image degraded to about 36 μm instead of 10 μm at the diffraction limit. This induced smoothing of the image was advantageous for an unambiguous extraction of the mirror topography when unwrapping the phase maps, because it bridged the gaps between mirror segments.

The mirror topography was obtained as follows:

1. Measurement of the interferogram between the reference beam and the beam from the deformable mirror.

**Figure 2:**
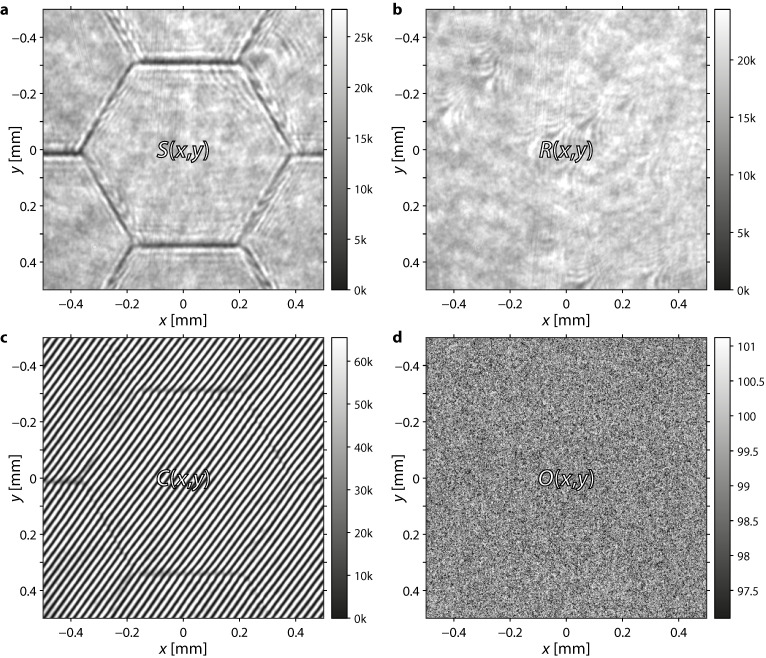
Illustration of the measured intensity images: (**a**) intensity reflected from the deformable mirror; (**b**) intensity reflected from the reference mirror; (**c**) interference pattern from both mirrors; (**d**) offset of the camera sensor.

The average bias value of each camera pixel was measured once with closed lid in front of the camera sensor. These offsets O(x,y) were then subtracted from all measured images further on (Figure 2). S(x,y), R(x,y), and C(x,y) are the pixel values in the sample, reference, and interference images, respectively, where nine frames were recorded and averaged for reduced measurement noise. The intensity $I_{s}\left(x,y\right)=S\left(x,y\right)-O\left(x,y\right)$ of the sample beam and the intensity $I_{r}\left(x,y\right)=R\left(x,y\right)-O\left(x,y\right)$ of the reference beam were measured by blocking the other beam with a shutter. The interference pattern $I_{c}\left(x,y\right)=C\left(x,y\right)-O\left(x,y\right)$ was measured by opening both shutters.

**Figure 3:**
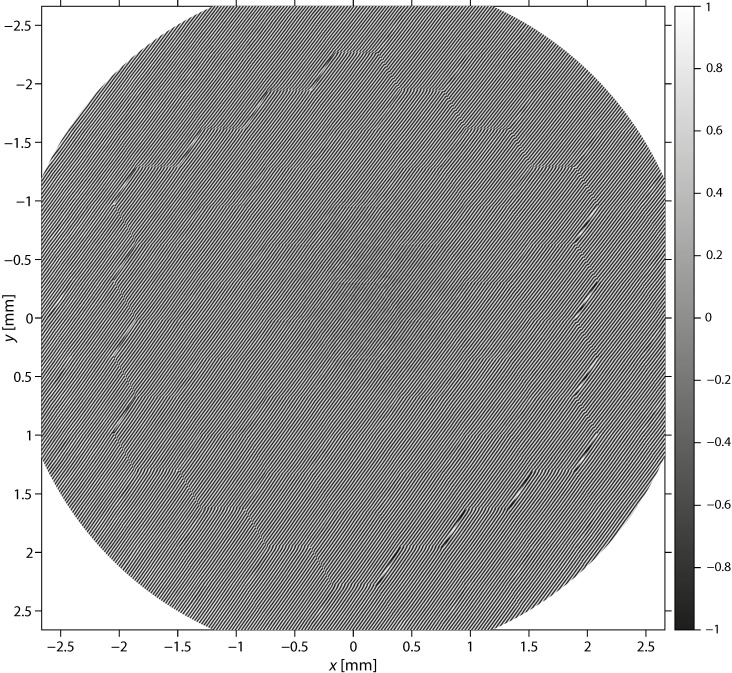
Interferogram of the deformable mirror in its full extent with corners excluded from further analysis.

The interferogram (Figure 3) was calculated by

(1) $$I\left(x,y\right)=\max\left\{-1,\min\left\{\frac{I_{c}-I_{r}-I_{s}}{2\sqrt{\max\left\{1,I_{r}I_{s}\right\}}},1\right\}\right\}$$

Its values were limited to the range [−1, 1]. The square root argument was lower bound to 1 to avoid potential issues with dark spots in the intensity images.

2. Demodulation of the interferogram to obtain the phase map [18,19].

The measured interferogram was limited to a circle of a diameter 1.1× the extent X×Y of its sides to eliminate its barely lit corners (Figure 3). The tilted reference beam introduced a modulation of the interferogram with a spatial frequency $\left(K_{x},K_{y}\right)$. This frequency was extracted from an interferogram measured with a flat reference mirror (Thorlabs BB2-E02) by locating the beating maxima in the spatial spectrum of the interferogram. The spatial spectrum $ℐ\left(k_{x},k_{y}\right)$ of the interferogram was recentered at this modulation frequency.

(2) $$ℐ\left(k_{x},k_{y}\right)=\overset{}{\underset{x^{2}+y^{2}<0.15\left(X^{2}+Y^{2}\right)}{\iint }}I_{c}\left(x,y\right)\exp\left(-ix\left(k_{x}+K_{x}\right)-iy\left(k_{y}+K_{y}\right)\right)dxdy$$

**Figure 4:**
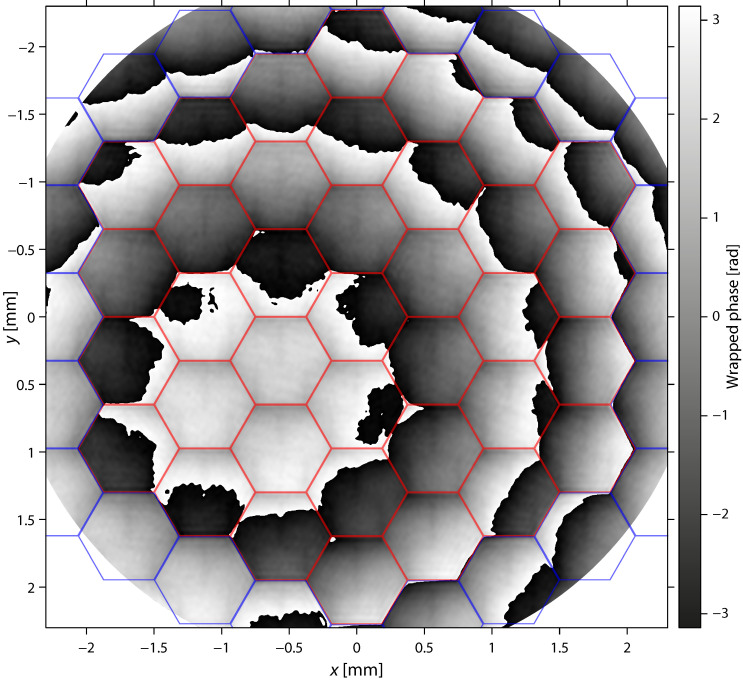
Wrapped phase map ϕ(x,y). Red hexagons indicate the active segments, and blue hexagons indicate the inactive segments of the deformable mirror.

The aperture limited the spatial spectrum to frequencies smaller than the modulation frequency. The demodulated interferogram was therefore calculated by the inverse Fourier transform of the limited spectrum, and its argument was taken to obtain the phase map (Figure 4).

(3) $$\phi \left(x,y\right)=\arg\left(\overset{}{\underset{k_{x}^{2}+k_{y}^{2}<0.49\left(K_{x}^{2}+K_{y}^{2}\right)}{\iint }}ℐ\left(k_{x},k_{y}\right)\exp\left(ixk_{x}+iyk_{y}\right)dk_{x}dk_{y}\right)$$

3. Unwrapping and scaling of the phase map to obtain the raw topography.

The phase map obtained in step 2 was wrapped (modulo 2π). We implemented the unwrapping algorithm proposed by Herráez et al. [20] to remove 2π phase steps. The edges between the active and inactive mirror segments were marked as loosely connected, forcing the algorithm to unwrap these regions with least preference. The unwrapping resulted in a continuous phase map Φ(x,y) that might still hide phase steps of multiples of 2π at the mirror segment edges, in particular, between the active and inactive segments. Fortunately, the limited spatial resolution of the interferogram eliminated discontinuities in the unwrapped phase map between the active segments for smooth deformations.

The raw topography was obtained by scaling the phase map with the wavelength λ.

(4) $$z_{s}\left(x,y\right)=\frac{\lambda }{4\pi }\Phi \left(x,y\right)$$

4. Correction of the optical system aberrations and removal of the mirror tip and tilt.

The imaging telescope introduced residual uncompensated aberrations between the sample and reference beams. Therefore, we measured the topography $z_{r}\left(x,y\right)$ of a flat mirror (Thorlabs BB2-E02) placed as a sample and subtracted it from the sample topography $z_{s}\left(x,y\right).$

(5) $$z\left(x,y\right)=z_{s}\left(x,y\right)-z_{r}\left(x,y\right)$$

The residual tip and tilt of the deformable mirror was finally removed by least squares fitting a flat reference surface to the inactive segments in the periphery of the measured topography image.

(6) $$\left\{\hat{a},\hat{b},\hat{c}\right\}=\underset{a,b,c}{\mathrm{argmin}}\overset{}{\underset{\left(x,y\right)\;\in \;\mathrm{inactive}\;\mathrm{area}}{\iint }}{\left(z\left(x,y\right)-ax-by-c\right)}^{2}dxdy\;$$

**Figure 5:**
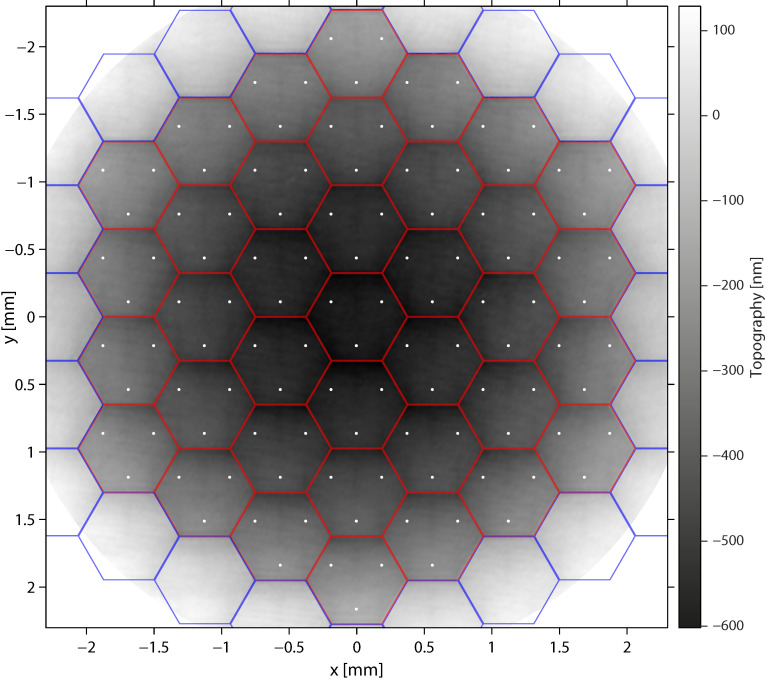
Referenced topography Δz(x,y) obtained from the phase map shown in Figure 4. Red hexagons indicate the 37 active segments of the deformable mirror and white dots the attachment points of its 111 actuators. Blue hexagons outline inactive mirror segments.

The referenced topography Δz(x,y) was then obtained by removing the reference surface (Figure 5).

(7) $$\Delta z\left(x,y\right)=z\left(x,y\right)-\hat{a}x-\hat{b}y-\hat{c}$$

The reference mirror in the sample arm was measured repeatedly to verify the precision of our method. The measured topography was within ±1.0 nm at 90% confidence and within ±1.6 nm at 99% confidence. The average RMS measurement error was 0.6 nm (<*λ*/1000).

## 3. Results

With the interferometer, we first measured the mirror topography for many actuator displacements. We then used the obtained calibration data to control the mirror into different shapes and verified the fidelity of these shapes using the interferometer again.

### 3.1. Calibration

The deformable mirror was adjusted in the sample arm such that the image of its active segments was centered on the camera sensor. The mirror’s axial position was adjusted for the sharpest image, and its main reflection was aligned to pass through the aperture near the optical axis of the imaging system. Images of the deformable mirror’s segments were mapped to the mirror segments. We traced the edges of the active area for the loose phase unwrapping in step 3 of the interferogram analysis.

As we encountered issues with the identification of the actuators, each actuator was commanded individually to its maximum voltage, and the mirror topography was analyzed to identify its position.

Next, we measured the displacements of the actuators when commanded by equal voltages. The voltages were increased in 256 steps from zero to the maximum, and the topography of the mirror was measured for each voltage. Due to the loose phase unwrapping at the edges of the active area, the measured topography included discontinuities there. These discontinuities were removed by adding multiples of λ/2 to the measured topographies of the active area. The topography of each active segment was fitted to a flat surface. The actuator displacements were obtained by these surfaces’ values at the actuators’ positions.

**Figure 6:**
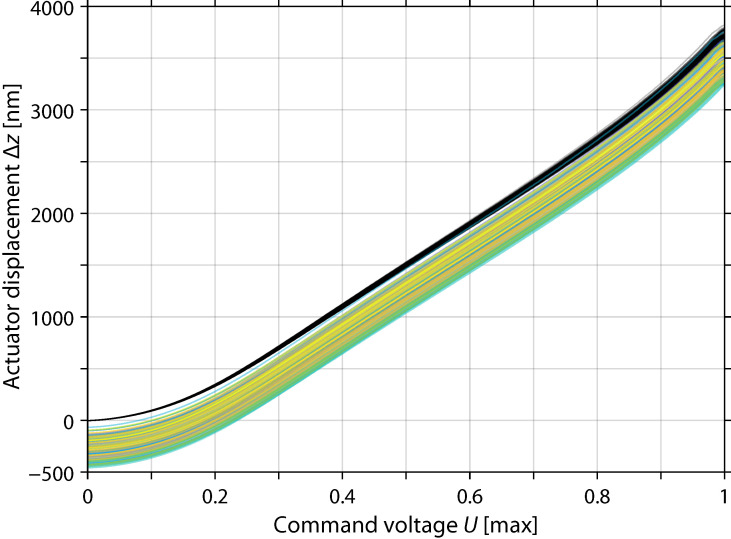
Actuator displacements versus command voltage. The colored curves show the absolute displacements extracted from the mirror topographies. The black curves show the displacements relative to zero voltage.

The obtained calibration data contained the coordinated displacements of all actuators over the full stroke (Figure 6). Command voltages of 20% to 80% of the maximum resulted in an approximately linear action on the mirror segments. The actuators showed similar typical relative displacements with about a 1% RMS scale error corresponding to a ±35 nm RMS displacement error at the maximum command voltage.

We fitted the displacement curve of each actuator i to the following model for U∈[0, 1]:

(8) $$\Delta z_{i}\left(U\right)=\left(\sqrt[{\alpha_{i}}]{1+\beta U^{\alpha_{i}}}-\overset{11}{\underset{n=0}{\sum }}\delta_{i,n}U^{n}\right)\mu m$$

The root terms and the constant of the polynomial modeled the actuator displacements for U<0.9, where $\alpha_{i}\approx 2.20$ to 2.25, β≈30, and $\delta_{i,0}\in \left(1,1.5\right)$. The eleventh-order polynomial refined the model. In particular, it accounted for the nonlinearity at mid-range and full stroke. The fit residuals stayed at about 1 nm RMS for U<0.92 but increased up to 10 nm at full stroke, because some actuators reached their maximum displacement for U>0.95.

**Figure 7:**
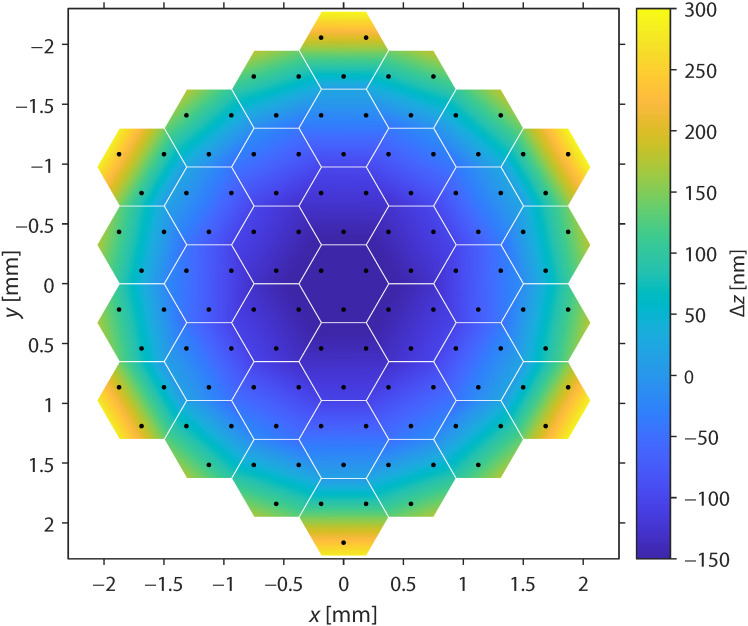
Average shape at equal command voltages for all actuators of the deformable mirror.

The mirror showed a parabolic surface curvature with about 450 nm peak-to-valley displacement among the active segments (Figure 7). If the reference shape of the mirror must be flat, the active curvature correction eats into the useful stroke of the deformable mirror. However, when the parabolic mirror surface was compensated by a defocusing of the optical system, the useful stroke was reduced by less than 100 nm. In this case, up to 3.6 μm remained available.

### 3.2. Open-Loop Actuator Control

The measured displacement curves for each actuator i were used as look-up tables to find the command voltage $U_{i}\left(\Delta z_{i}\right)$ of the requested displacement by linear interpolation. We tipped and tilted the entire active surface and adjusted the commanded displacements until smooth surfaces were measured. The actuator triplets of each segment required an overdrive by 24% of their differential displacements to compensate for the mechanical crosstalk. No crosstalk was observed between the segments.

We commanded the deformable mirror to shapes that would be required for moving the beam focus in a laser beam-scanning microscope in three dimensions. We used a clear aperture diameter of $\emptyset_{\mathrm{dm}}=3.6$ mm of the deformable mirror that was fully covered by the active segments. The objective has a focal length $f_{\mathrm{obj}}$, a numerical aperture NA, and an immersion medium with refraction index n. The deformable mirror topography for displacing the beam focus by (X,Y,Z) is a linear combination of basic shapes,

(9) $$\Delta z\left(x,y\right)=X\;\Delta z_{X}\left(x,y\right)+Y\;\Delta z_{Y}\left(x,y\right)+Z\;\Delta z_{Z}\left(x,y\right)+\Delta z_{0}\left(x,y\right),$$

where $\Delta z_{X}$, $\Delta z_{Y}$, and $\Delta z_{Z}$ are the characteristic shapes for tip, tilt, and defocus, respectively. $\Delta z_{0}$ is the reference displacement. It corrects the aberrations of the deformable mirror and the optical system.

**Figure 8:**
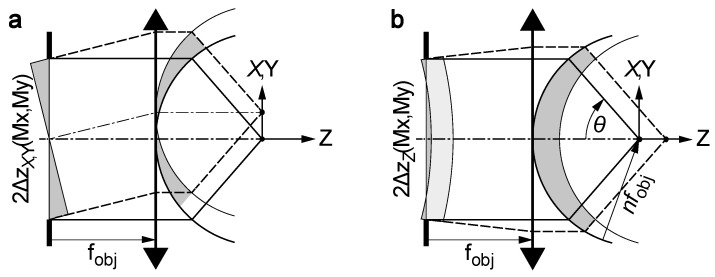
Illustration of the wavefront deformation for shifting the focus (**a**) in the lateral plane and (**b**) along the optical axis. The objective is simplified as a thin lens with flat principal plane on the aperture side, coinciding with the lens, and spherical principal plane on the object side, centered on the focus. An offset (light gray) is trimmed from the characteristic shape $\Delta z_{Z}$ (dark gray) to obtain minimal displacements.

The deformable mirror was imaged into the aperture of the objective, whose diameter is $\emptyset_{\mathrm{obj}}=2\mathrm{NA}f_{\mathrm{obj}}$. The lateral displacement was proportional to the tip and tilt of the wavefront in the aperture, which was twice the tip and tilt of the mirror surface divided by the magnification $M=\emptyset_{\mathrm{obj}}/\emptyset_{\mathrm{dm}}$ of the relay (Figure 8a).

(10) $$\Delta z_{X}\left(x,y\right)=\frac{x}{2Mf_{\mathrm{obj}}}=\frac{\emptyset_{\mathrm{dm}}}{4f_{\mathrm{obj}}^{2}\mathrm{NA}}x$$

(11) $$\Delta z_{Y}\left(x,y\right)=\frac{y}{2Mf_{\mathrm{obj}}}=\frac{\emptyset_{\mathrm{dm}}}{4f_{\mathrm{obj}}^{2}\mathrm{NA}}y$$

The defocus shape was calculated by observing that a shift Z of the focus along the optical axis requires a wavefront shift of nZcos(θ), where θ is the angle from the optical axis under which a point on the wavefront is seen from the focus (Figure 8b). As the objective fulfills Abbe’s sine condition, we note that $M\sqrt{x^{2}+y^{2}}=nf_{\mathrm{obj}}\sin\left(\theta \right)$.

(12) $$\Delta z_{Z}\left(x,y\right)=C-\frac{n\cos\left(\theta \right)}{2}=C-\sqrt{\frac{n^{2}}{4}-\frac{{\mathrm{NA}}^{2}}{\emptyset_{\mathrm{dm}}^{2}}\left(x^{2}+y^{2}\right)}$$

The constant C centered the displacements to minimize the required actuator stroke. Within the aperture of the objective, the argument of the square root remained positive.

The reference displacement $\Delta z_{0}\left(x,y\right)$ was set to the matched defocus $Z_{0}\;\Delta z_{Z}\left(x,y\right)$ that fitted the average shape of the deformable mirror. An offset was included to drive the actuators at about half-stroke for (X,Y,Z)=(0, 0, 0). An approximately flat topography was obtained for (X,Y,Z)=(0, 0, 1) μm, and ranges of about ±(2, 2, 3) μm could be addressed.

### 3.3. Validation

Each shape was measured and its topography analyzed by decomposing it in the three characteristic shapes of (10), (11) and (12), plus a flat offset. The measured deformations followed the commanded deformations to 19 nm RMS error in the beam position (X,Y,Z) and 12 nm median error. This error corresponded to 0.1–0.2% of the validated range. For all validated shapes, the measured RMS residuals fulfilled the manufacturer’s specification for a flat surface (<40 nm). The measured RMS residuals were mostly <20 nm, approaching the manufacturer’s calibration value of 16 nm for flat topographies in closed-loop control. As the RMS residual increased with decreasing Z, the aperture in our interferometer may not have been placed exactly in the common focal plane of the telescope, resulting in a focus-dependent extent of the spatial spectrum, and/or the deformable mirror and camera sensor were not placed in the corresponding focal planes of the telescope lenses.

**Figure 9:**
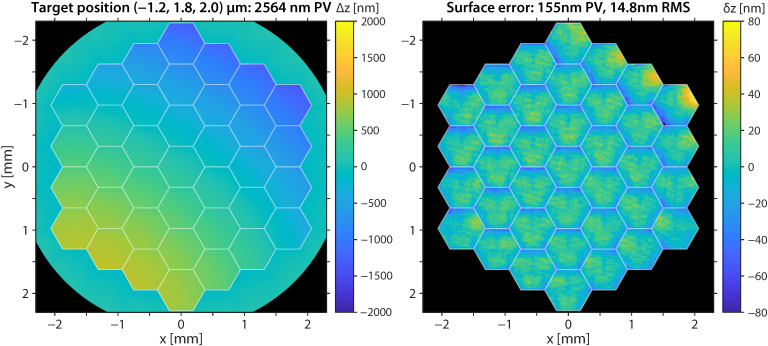
Illustration of a measured deformable mirror topography for positioning the laser beam focus at (−1.2, 1.8, 2.0) μm. The shape error was most pronounced at the minimal actuator displacement in the top-right corner.

Figure 9 and Supplementary Video S1 illustrate the measured deformable mirror topographies and the shape errors for steering the laser beam focus at different target positions (X,Y,Z). The shape errors were most pronounced on the segments whose actuator(s) were driven close to the minimum or maximum voltage. There, the open-loop calibration could not fully compensate the nonlinear actuator responses. The crosstalk between the actuators of one segment may be a major cause for the observed deviations.

**Figure 10:**
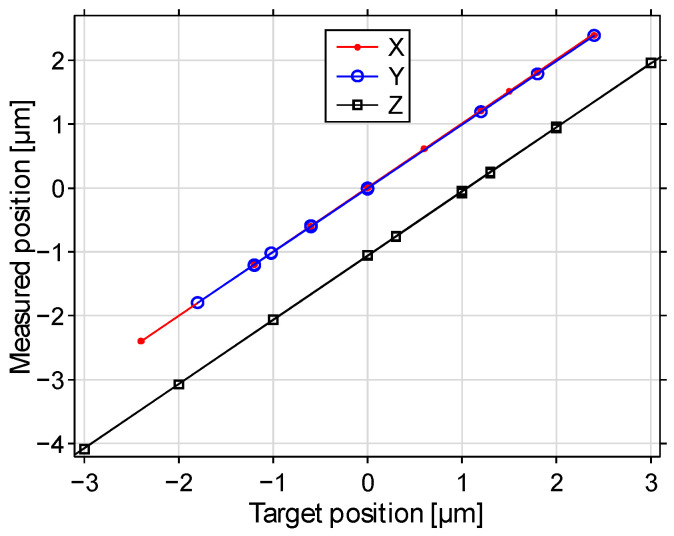
Measured positions versus target positions (X,Y,Z). The axial position is biased by the reference defocus.

**Figure 11:**
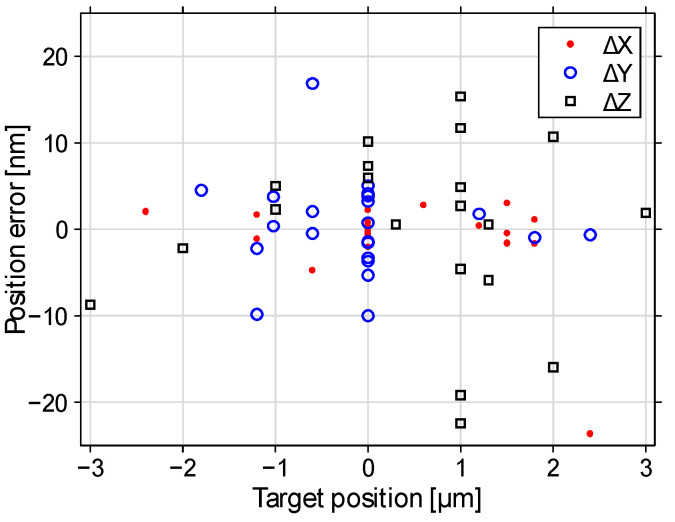
Position errors versus target position (X,Y,Z). Most individual positioning errors are within ±10 nm.

**Figure 12:**
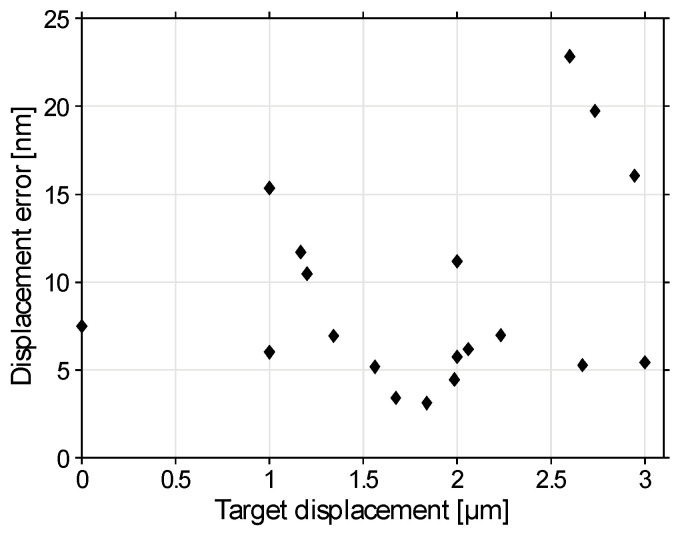
Displacement error of the beam focus versus target displacement $\sqrt{X^{2}+Y^{2}+Z^{2}}$. The average measured displacement error was <0.6% of the displacement.

Figure 10, Figure 11 and Figure 12 illustrate the beam positioning accuracy and precision for various target positions. Laterally, a field of X,Y∈(−2.5, 2.5) μm was addressed. Up to Z∈(−3, 3) μm were addressable axially, shifted by about −1 μm by the surface curvature of the mirror that we chose not to correct. The measurement uncertainty contributed 5 to 7 nm to the measured positioning error. Regardless, the positioning errors remained mostly within ±10 nm deviation. The errors were more pronounced if some actuators approached their minimum and/or maximum displacements, where the displacement curves showed significant nonlinearity.

## 4. Discussion and Conclusions

Although we obtained a precision of about 1 nm, the accuracy of our measurements was limited by the surface flatness of the reference mirror (*λ*/10 on 50 mm diameter) and its scratch-dig (10-5).

Most commonly, dielectric mirrors are bent spherically due to the mechanical stress between the coating and the substrate. As a spherical deformation scales with the square of the diameter, we assumed a surface flatness of about *λ*/1000 on the 5 mm diameter that was used. At the specified scratch-dig, the reference mirror may have several scratches of at most 10 μm width for up to a total length of 12.5 mm. It may have up to five maximum-sized digs of 0.05 mm, and the sum of the diameters of all digs must not exceed 0.5 mm. We did not observe any visible scratches or digs.

When moving the reference mirror laterally, the measured reference topographies differed by less than ±2 nm, which we deemed acceptable. For our application, we could accept larger differences because they could be removed together with other aberrations of the optical system by finding the resting shape of the deformable mirror that maximized the optical performance of the system.

The model Equation (8) was used to interpolate the actuator responses in between the measured points. We chose the lowest polynomial order yielding fit residuals of less than about 1 nm to the data. A higher polynomial order would introduce undesired excursions in between the measured samples for matching these more closely, and it would suppress the measurement noise less effectively.

The calibration of the deformable mirror topography for each actuator over its full displacement range allowed controlling the mirror with a precision and accuracy in an open-loop operation that would normally require a closed-loop operation. The measurement of the mirror topography also enabled accounting for the crosstalk among actuators of the same mirror segment. Some oversteering was necessary to achieve the desired tip and tilt of the mirror segments.

We performed calibration measurements several times in February and June and observed differences of 7–8 nm RMS between the actuator displacements curves. These differences were highly correlated among all actuators and were attributed to residual changes in the tip–tilt and surface curvature of the deformable mirror. The full stroke differed by about 7 nm (0.2% scale change), and the phase unwrapping caused 3 nm uncertainty near the 12 phase steps over the full stroke. A validation of the calibration measurement three weeks later increased the residual RMS shape error by 2–3 nm over the immediate validation. As the temperature of the driver electronics may affect the actuator responses, we let all devices warm up on standby for at least half an hour. However, transient thermal effects caused by alternating dynamical use of the deformable mirror may further increase its residual shape error and cause a scale error in beam steering applications.

The calibrated deformable mirror featured a field of about twice the range of an electro-optic deflection system laterally [10] and about ten times the range of an electro-optic lens axially [21]. Beam positioning with the deformable mirror was somewhat faster (40–200 μs) than with galvanometer mirror scanners (100–300 μs) but significantly slower than with electro-optic scanners (<1–10 μs). The positioning accuracy was similar to galvanometer mirror scanners (10 nm) but worse than electro-optic scanners (1 nm). However, the correction for optical aberrations of the microscope system and the sample is a valuable benefit of the deformable mirror. With other scanners, aberration correction would require dedicated optical elements.

In conclusion, the deformable mirror featured an attractive combination of rapid and accurate beam positioning in a sample within an extended field as compared to electro-optic scanners. The calibration of all its individual actuators improved their positioning and thereby the accuracy of the mirror’s shape in open-loop control.

## Figures and Tables

**Table 1:** Comparison of the considered wavefront measurement methods and calibration data.

Method	Sub-Aperture PSF Imaging		Off-Axis Imaging Interferometry	
**Instrument**	Shack–Hartmann sensor	Plenoptic sensor, light field camera	Common-path quantitative phase imager	Imaging Michelson interferometer
**Sampling points** (4 Mpixel sensor)	$\sim 10^{4}$	$\sim 10^{5}$	$\sim 10^{6}$	$\sim 10^{6}$
**Measured** **quantity**	Mean wavefront gradients on sub-apertures	Wavefront gradients on sub-apertures	Wavefront versus low-frequency self-reference	Wavefront versus reference beam
**Conditions for wavefront reconstitution**	Continuous smooth wavefront	Continuous wavefront	Localized wavefront perturbations	Requires a known reference wavefront
**Issues for our task**	The sampling is too coarse. The quantification of the common actuator displacement is error prone.		Large features are underreported.	None
**Suitability**	Low	Low	Moderate	High
**Manufacturer** **calibration data**	Flat-field actuator control values $U_{i}\left(\bar{z}\right)$ at mid-range $\bar{z}$ and typical actuator displacement curve Δz(U).			
**Measured data**	Deformable mirror topographies z(x,y,U) for actuator control values U over full range and mirror segments and actuator positions for $z\left(x,y,U\right)\to z_{i}\left(U\right)$.			
**Required data**	Actuator control values $U_{i}\left(z_{i}\right)$ for target placements $z_{i}$ over full stroke.			

## Data Availability

Sample data and analysis tools can be obtained from the authors upon reasonable request.

## Supplementary Materials

The following supporting information can be downloaded at: https://www.mdpi.com/article/10.3390/s22218465/s1, Video S1: Illustration of measured deformable mirror topographies and residual errors when steering the laser beam focus at several target positions.
